# CD36 regulates lipopolysaccharide-induced signaling pathways and mediates the internalization of *Escherichia coli* in cooperation with TLR4 in goat mammary gland epithelial cells

**DOI:** 10.1038/srep23132

**Published:** 2016-03-15

**Authors:** Duoyao Cao, Jun Luo, Dekun Chen, Huifen Xu, Huaiping Shi, Xiaoqi Jing, Wenjuan Zang

**Affiliations:** 1 Shaanxi Key Laboratory of Molecular Biology for Agriculture, College of Animal Science and Technology, Northwest A&F University, Yangling, 712100, PR China

## Abstract

The scavenger receptor CD36 is involved in pathogen recognition, phagocytosis, and pathogen-induced signaling. This study investigated the relationship between CD36 and TLR4 in modifying lipopolysaccharide (LPS)-induced signaling pathways and mediating *Escherichia coli* (*E. coli*) endocytosis in primary goat mammary epithelial cells (pGMECs). The manipulation of CD36 expression significantly influenced TLR4 and nuclear factor kappa B (NF-κB) mRNA expression in pGMECs stimulated with LPS for 12 h. NF-κB and activator protein-1 (AP-1) activity was regulated by the manipulation of CD36 expression in LPS-induced pGMECs. However, CD36-mediated AP-1 activation occurred primarily through c-Jun N-terminal kinase (c-JNK). Adaptor proteins and proinflammatory cytokines were also involved in these signaling pathways and acted by regulating CD36 expression in LPS-stimulated cells. Moreover, CD36 cooperated with TLR4 in TLR4-mediated phagocytosis following *E. coli* simulation, but this complex was not induced by LPS treatment. Our study is the first to illuminate CD36 as a scavenger receptor in ruminants. Additionally, this study indicates that CD36 plays a vital role in the LPS-induced activation of downstream signaling cascades and mediates *E. coli* phagocytosis *via* TLR4 in pGMECs, which offers a novel treatment strategy for mastitis.

*Escherichia coli* (*E. coli*) is one of the most severe pathogens in epidemiology and is the most common bacteria to cause mastitis in dairy herds. Mastitis is a worldwide disease that not only inflicts economic damage on the dairy industry but also threatens consumer health^1^,^2^. In mastitis, mammary epithelial cells (MECs) become infected with *E. coli*. During the infection, the cells engulf the pathogens and secrete cytokines to activate immune cells, such as phagocytes and granulocytes^3^. In response to *E. coli*, MECs produce a variety of cytokines and initiate a proinflammatory response themselves primarily through toll-like receptor 4 (TLR4)^4^. After the onset of an *E. coli* infection, TLR4 assembles the TLR4/MD-2/LPS complex on the surface of the cell and initiates a downstream signaling pathway to induce the production of proinflammatory mediators to eliminate the bacteria^5^. LPS induces the co-clustering of TLR4 with CD14 and with chemokine receptor 4^6^, Fcγ receptors^7^, and scavenger receptors (SRs)^8^. Some of these proteins can function as CD14, such as class B scavenger receptors, including CD36, CLA-1/SRB-I, and CLA-2/SRB-II^9^,^10^. However, which receptors participate in or cooperate with TLR4 during *E. coli* recognition and LPS initiation remains unknown.

CD36 is a membrane glycoprotein present in platelets, mononuclear phagocytes, adipocytes, hepatocytes, myocytes, and some epithelial cells^11^. Depending on its distribution, this protein is also involved in the uptake of apoptotic cells and in the modulation of inflammation, atherosclerosis, diabetes, and cardiomyopathy^10^. Some researchers have found that CD36 plays a role in the internalization of Gram-positive and Gram-negative bacteria as well as in LPS recognition during the early stages of infections^9^,^12^. In recent studies, CD36 was shown to be recruited to TLR2/TLR6 heterodimers to form a CD36/TLR2/TLR6 complex in lipoteichoic acid (LTA) and diacylated peptide stimulation^13^,^14^. CD36 can not only invoke cell secretion of cytokines in pathogen-induced signaling but also play vital roles in bacterial phagocytosis and clearance^12^,^15^. Moreover, some studies have shown that cells lacking phagocytic abilities will acquire or increase phagocytic functions following transfection with CD36^16^,^17^. These reports revealed that CD36 is also capable of activating cells alone or in conjunction with other receptors to recognize danger signals, thereby eliminating potential “self” or “nonself” threats. Most studies are interested in phagocytic cells; however, little is known about the function of CD36 in nonphagocytic cells, such as epithelial cells, which act as a “sentinel” to guard mammary tissue from exogenous threats. Therefore, understanding the role of CD36 in MECs during an infection is important.

Nuclear factor-kappa B (NF-κB) and activator protein-1 (AP-1) are the most important transcription factors in the TLR family of receptors that induce inflammation^18^,^19^. After ligand stimulation, the interleukin-1 receptor-associated kinase (IRAK) family (IRAK1, 2, and 4) is activated and has an essential role in the activation of NF-κB and mitogen-activated protein kinase (MAPK) downstream of MyD88. IRAK activation results in an interaction with tumor necrosis factor (TNF) receptor-associated factor 6 (TRAF6) to activate TAK1 downstream^20^. TAK1 can phosphorylate IKKβ through its close proximity to the IKK complex (IKKα-IKKβ-IKKγ), which leads to NF-κB activation *via* the phosphorylation and subsequent degradation of IκB proteins^21^. TAK1 also simultaneously activates MAPKs [extracellular signal-related kinase 1 (ERK1), ERK2, p38, and c-Jun N-terminal kinase (c-JNK)] by inducing the phosphorylation of MAPK kinases, which then activate various transcription factors, including AP-1, and influence cytokine production^22^. The mechanisms of TLR4 activation and downstream signaling cascades are gaining attention from veterinary researchers. A recent finding suggested that TLR4 expression is upregulated and activated downstream of NF-κB, JNK, and ERK in a mouse model of LPS-induced mastitis^23^. Bovine MECs (bMECs) stimulated with *E. coli* or LPS revealed that NF-κB plays a vital role in *E. coli*- or LPS-induced infections through TLR4-induced inflammation^24^. Interestingly, CD36, acting as a scavenger receptor, can also participate in the bacteria-induced inflammation process and activate downstream signaling pathways. In Bacillus Calmette-Guérin (BCG)-infected macrophages, CD36 participates in lipid accumulation and downmodulates inflammatory responses through peroxisome proliferator-activated receptor gamma (PPARγ)-dependent and NF-κB-independent pathways^25^. CD36-deficient mice exhibit reduced levels of activated NF-κB and oxidative stress, indicating that CD36 is a key mediator of proinflammatory and oxidative signaling pathways^26^. Recent data revealed that CD36 mediates the activation of Src kinases (Fyn and Lyn) followed by the downstream activation of JNK and ERK1/2 kinases^27^,^28^. The proinflammatory response and bacteria uptake were also shown to be dependent on CD36-JNK signaling^12^. However, little is known about the effect of CD36 on downstream signaling pathways in mastitis because most studies have focused on the role of CD36 as a lipid transporter (FAT/CD36) and have neglected its role as a scavenger receptor in dairy ruminants. The present study determined the characteristics of CD36 during a LPS-induced inflammatory response and examined whether CD36 cooperates with TLR4 in pathogen phagocytosis in primary goat MECs (pGMECs). The primary goal of this study was to test the hypothesis that CD36 may be involved in LPS-induced inflammation *via* the NF-κB and JNK signaling pathways and accompany TLR4 during TLR4-mediated *E. coli* endocytosis in pGMECs.

## Results

### Activation of TLR4 and CD36 in *E. coli*-induced mastitis

**Figure 1:**
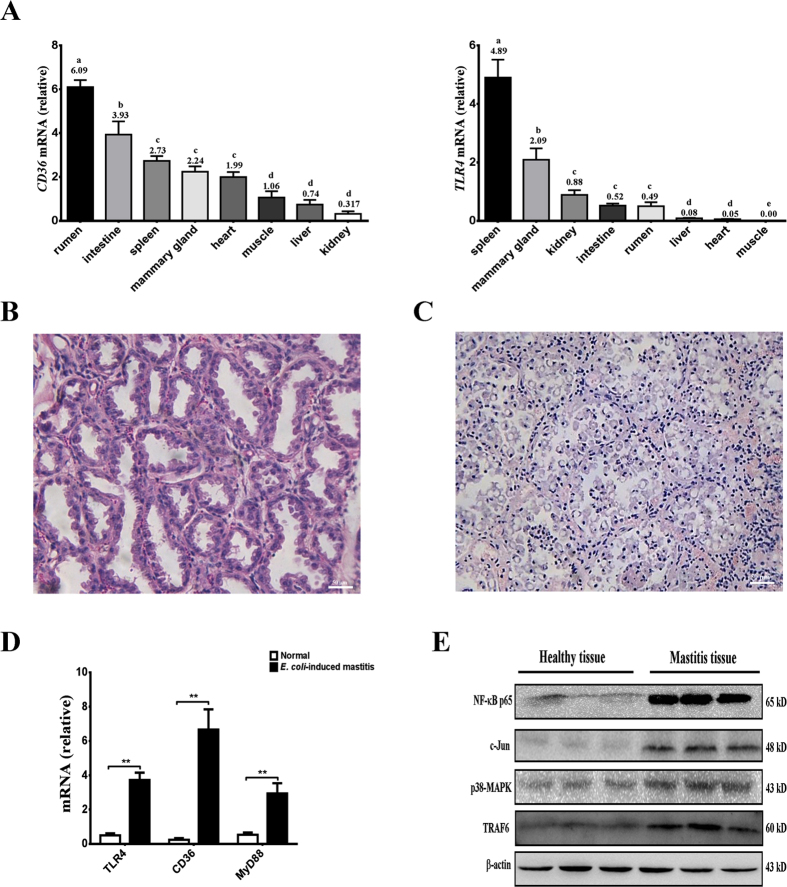
Distribution of CD36 and TLR4 expression in various tissues from dairy goats following *E. coli*-induced mastitis. (**A**) Relative distribution of CD36 and TLR4 mRNA expression in different goat tissues. The relative expression values are shown on the top of each bar. (**B**,**C**) Histology of the dairy goat mammary gland. In healthy dairy goats, epithelial tight junctions, intact acinar structures, and no infiltrated inflammatory cells are observed in the mammary tissue of the uninfected udder (**B**, left). Acinar epithelial cells and inflammatory cells infiltrated the acinar lumina, causing interstitial edema, and the acinar structures disintegrated in *E. coli*-induced mastitis (**C**, right). (**D**) Relative to the healthy dairy goat, the mRNA expression of CD36, TLR4, and MyD88 increased significantly in *E. coli*-infected goats (**P < 0.01). (**E**) NF-κB, c-JUN, p38-MAPK, and TRAF6 protein levels were enhanced in mastitis tissues compared to health tissues. The values are the means ± SEM for three individuals. Quantitative PCR data were normalized to GAPDH, UXT, and MRPL39.

TLR4 and CD36 are important receptors for recognizing pathogens and activating downstream signaling; however, little is known about TLR4 and CD36 in dairy animals, especially in dairy goats. Therefore, the present study first detected the distribution of TLR4 and CD36 in healthy dairy goats to understand the expression status in different tissues, particularly in the mammary gland (Fig. 1A). To understand the distribution of CD36 and TLR4 in dairy goats, mRNA was collected and extracted from six healthy dairy goats (during the lactation period) to detect CD36 and TLR4 mRNA levels in various tissues, particularly those tissues that play important roles in metabolism and immune functions. CD36 mRNA expression levels were the highest in the rumen (*P* < 0.05) compared with other tissues. TLR4 mRNA levels were expressed at higher levels in the spleen (*P* < 0.05). Both CD36 and TLR4 were moderately expressed in the mammary gland tissues. Interestingly, CD36 and TLR4 mRNA levels were the lowest in the kidney and muscle, respectively (Fig. 1A). To confirm *E. coli*-induced mastitis, milk samples were analyzed, and the bacteria were separated before the experiment (see Supplementary Fig. S1A,B and Table S1). Mammary gland tissue samples were collected from three *E. coli*-infected goats and three healthy dairy goats. Microscopic examination showed that the interstitium was infiltrated with inflammatory cells following infection (Fig. 1C). Compared with the control group, the acinar lumina and acinar structure of the infection group were disrupted by *E. coli* invasion, and the bacteria destroyed the epithelial tight junctions (Fig. 1B,C). TLR4 and MyD88 mRNA levels were higher in infected goats than in healthy goats (*P* < 0.01). Surprisingly, CD36 mRNA levels were also significantly increased in *E. coli*-induced mastitis compared with healthy goats (*P* < 0.01) (Fig. 1D). Unfortunately, changes in the CD36 and TLR4 proteins could not be detected because no suitable anti-goat antibodies are currently available. However, downstream signal changes revealed that NF-kB-p65, c-JNK, p38-MAPK, and TRAF6 were activated in *E. coli*-induced mastitis tissue samples compared with normal tissue samples (Fig. 1E). The results indicated that *E. coli*-induced mastitis could trigger TLR4 and CD36 mRNA expression and activate the downstream signaling pathways in Xinong Saanen dairy goats.

### CD36 participates in LPS-induced inflammation in pGMECs

**Figure 2:**
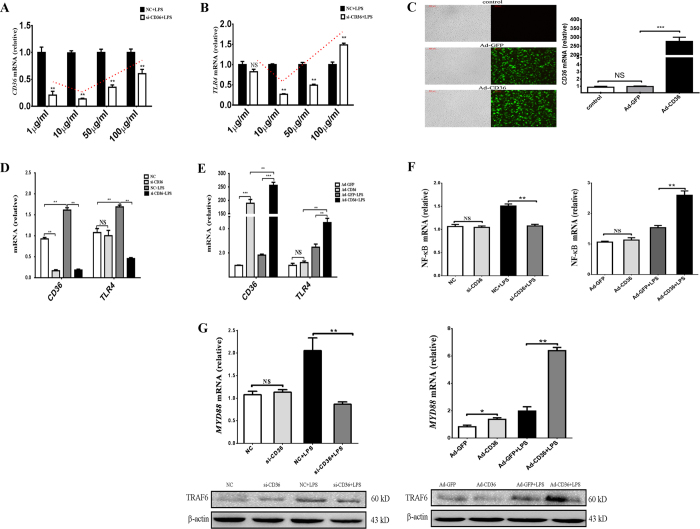
CD36 participates in LPS-induced inflammation in pGMECs. (**A**,**B**) Changes in CD36 and TLR4 mRNA levels are shown. Cells were pretreated with NC or si-CD36 for 24 h and then treated with LPS for 12 h. CD36 and TLR4 mRNA levels increased following the addition of various concentrations of LPS. (**C**) Bright field and fluorescence images of pGMECs infected with Ad-GFP (left, upper) and Ad-CD36 (left, lower) adenovirus for 24 h (MOI = 100). The changes in CD36 mRNA levels compared with the control group, Ad-GFP group, and Ad-CD36 group are shown. (**D**,**E**) CD36 and TLR4 mRNA changes in pGMECs pretreated with siRNA or adenovirus and then exposed to 10 μg/ml LPS for 12 h. (**F**) The variation in NF-κB mRNA levels following the manipulation of CD36 in pGMECs. NF-κB mRNA levels were influenced by knockdown (left) or overexpression (right) of CD36 in pGMECs, which were then stimulated with 10 μg/ml LPS. (**G**) MyD88 mRNA levels and TRAF6 protein levels were influenced by CD36 expression in LPS-stimulated pGMECs. The values are the mean ± SEM for three individuals. Quantitative PCR data were normalized to GAPDH, UXT, and MRPL39. All data are presented as the mean ± SEM from three experiments. *P < 0.05, **P < 0.01, ***P < 0.001, and not significant (NS).

The *in vivo* studies demonstrated that the CD36 receptor was involved in *E. coli*-induced mastitis. To understand the functions and roles of CD36 during the infection, *E. coli* LPS-induced inflammation in the pGMEC model was used to simulate the *in vitro* experiment. At concentrations of 1–10 μg/ml, LPS did not induce cell apoptosis or necrosis (see Supplementary Fig. S2A–D) but did trigger CD36 and TLR4 expression in LPS-stimulated cells (See Supplementary Fig. S2E,F). CD36 and TLR4 mRNA were detected at different LPS concentrations (1, 10, 50, and 100 μg/ml) before treating the cells with small interfering RNA (siRNA: si-CD36) for 24 h (Fig. 2A,B). Interestingly, the variation in CD36 mRNA levels was similar to that in TLR4 mRNA levels after adding LPS at different concentrations (Fig. 2A,B). From the above results, 10 μg/ml is the ideal LPS concentration to upregulate the expression of both CD36 and TLR4 mRNA in treated groups after 12 h.

CD36 mRNA levels were significantly increased (*P *< 0.001) in cells incubated with CD36 adenovirus (Ad-CD36) for 24 h, but no significant changes were noted between the control group and the group incubated with adenovirus (Ad-GFP) for 24 h (*P* > 0.05) (Fig. 2C). In contrast to the LPS-stimulated negative control (NC) or Ad-GFP groups, the CD36 and TLR4 mRNA levels declined dramatically in the deficiency groups (*P* < 0.01) (Fig. 2D), whereas their mRNA levels enhanced markedly in the Ad-CD36 cells (*P* < 0.01) (Fig. 2E). The NF-κB mRNA levels were also influenced by the manipulation of CD36 expression during the LPS-induced inflammation of pGMECs; however, no change was noted in si-CD36 or Ad-CD36 cells without LPS stimulation, indicating that CD36 could not invoke NF-κB activation alone in pGMECs without LPS stimulation (Fig. 2F). After incubating the cells with LPS for 12 h, MyD88 and TRAF6 were increased in the LPS-stimulated groups compared with the control groups (Fig. 2G). CD36 overexpression could enhance the MyD88 mRNA levels and TRAF6 protein expression in LPS-stimulated cells; however, the effects of both adaptor proteins were diminished in the si-CD36 group compared to the LPS-incubation group (Fig. 2G). According to the aforementioned results, CD36 works with TLR4 to regulate adaptor proteins (MyD88 and TRAF6) and activate the NF-κB downstream signaling pathway in LPS-induced inflammation in pGMECs.

### CD36 regulates NF-κB and c-JNK activation but not p38-MAPK kinase pathways following LPS stimulation in pGMECs

**Figure 3:**
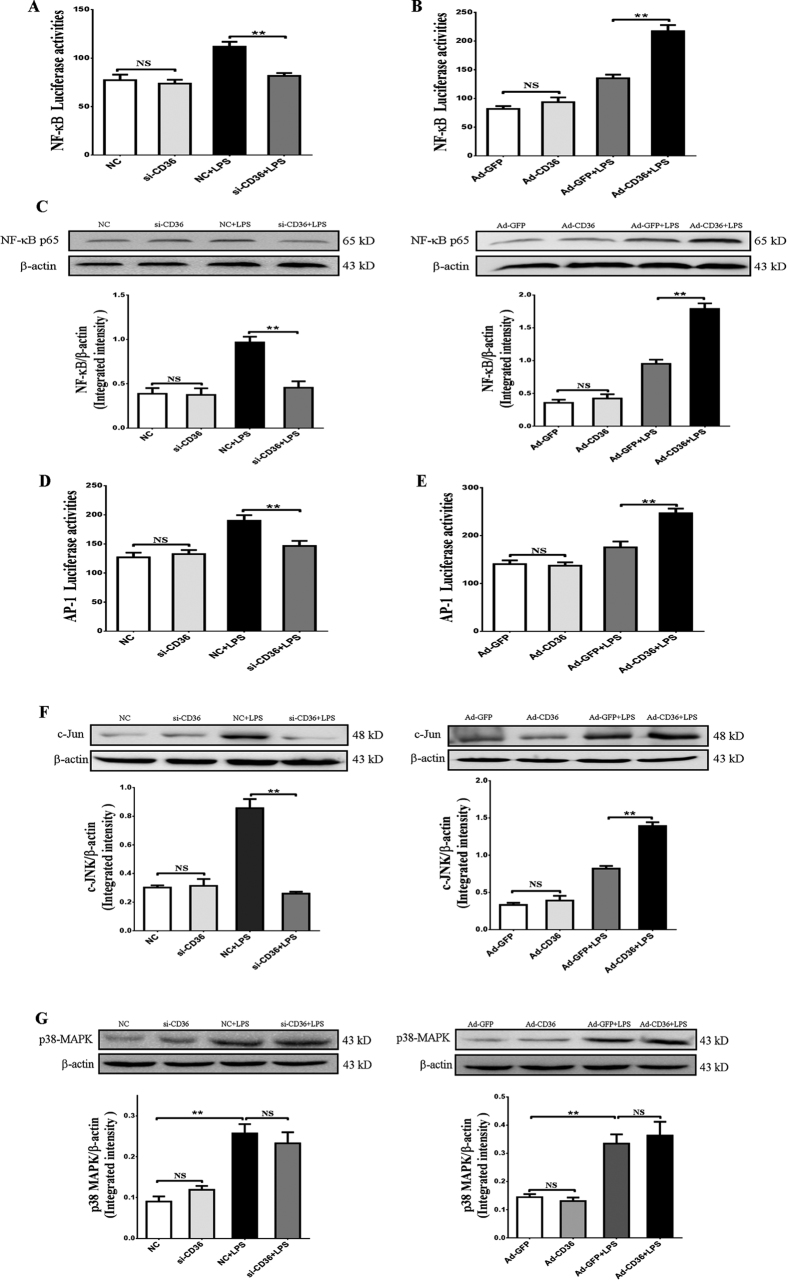
Manipulation of CD36 expression regulates NF-κB and JNK activation but not the p38-MAPK kinase pathway in pGMECs exposed to LPS stimulation. (**A**,**B**) The NF-κB-RE and *Renilla* luciferase vectors were cotransfected into pGMECs. Then, CD36 expression was manipulated followed by stimulation with LPS for 12 h, and the transcriptional activity of the NF-κB promoter was evaluated. (**C**) Overexpression or knockdown of CD36 expression on LPS-stimulated pGMECs influenced NF-κB p65 protein expression. (**D**,**E**) AP-1 luciferase activity was influenced by the CD36 expression status in LPS-stimulated cells. (**F**) c-Jun protein levels increased in Ad-CD36 cells and decreased in si-CD36 cells treated with LPS for 12 h. (**G**) p38-MAPK protein levels were detected in LPS-stimulated cells (Ad-CD36 or si-CD36). The corresponding mean gray values of NF-κB p65, c-Jun, and p38-MAPK protein levels were obtained from three separate blots. The graph shows the densitometric quantification of NF-κB p65/β-actin, c-Jun/β-actin, and p38-MAPK/β-actin as the fold change difference compared to the control. The relative mRNA levels of MyD88 were also detected. All data are presented as the mean ±SEM from three experiments. *P < 0.05, **P < 0.01, and not significant (NS).

The previous results showed that the knockdown or overexpression of CD36 could affect the NF-κB mRNA levels in pGMECs (Fig. 2F). Therefore, a NF-κB-RE luciferase reporter was transfected into the cells to evaluate the activation of the NF-κB signaling pathway in pGMECs. Adding LPS to CD36 knockdown pGMECs decreased NF-κB-RE luciferase activation significantly compared to the NC group (*P* < 0.01) (Fig. 3A). Pretreating cells with Ad-CD36 and then adding LPS increased the NF-κB-RE luciferase activation dramatically (*P* < 0.01) (Fig. 3B). Detection of the NF-κB protein revealed that CD36 knockdown in pGMECs decreased the protein levels and that CD36 overexpression increased the NF-κB protein significantly compared with the LPS-stimulated group (Fig. 3C). Previous studies have shown that LPS could also activate the AP-1 transcription factor *via* the TLR4 signaling pathway^29^,^30^. Therefore, we next investigated whether CD36 affected AP-1 activation during LPS-induced inflammation. Transfection of the AP-1-RE luciferase reporter in pGMECs demonstrated that AP-1 activation following LPS stimulation was influenced by the manipulation of CD36 expression in pGMECs. Cells stimulated with LPS showed increased AP-1 activation compared to the groups incubated without LPS (Fig. 3D,E). Treatment with si-CD36 before adding LPS revealed that AP-1 activation decreased significantly compared to the LPS-stimulated group (*P* < 0.01) (Fig. 3D). Compared with the GFP + LPS groups, AP-1 activation increased dramatically (*P* < 0.01) in Ad-CD36 cells stimulated with LPS (Fig. 3E). The aforementioned results indicated that CD36 participates in LPS-mediated AP-1 activation *via* the TLR4 signaling pathway. The AP-1 transcription factor could be activated by c-JNK and p38-MAPK. LPS-stimulated cells could increase c-JNK protein levels; moreover, adding Ad-CD36 to cells before LPS treatment led to a notable increase in c-JNK protein levels (*P* < 0.01) (Fig. 3F). However, the protein levels decreased considerably in the si-CD36 pretreatment groups compared to the LPS-stimulated groups (*P* < 0.01) (Fig. 3F). The p38-MAPK protein levels increased significantly after 12 h of LPS stimulation (*P* < 0.01) (Fig. 3G); however, cells treated with LPS and either Ad-CD36 or si-CD36 revealed that the difference in the expression of p38-MAPK between the two groups was not significant (*P* > 0.05) (Fig. 3G). Therefore, these results demonstrate that manipulating CD36 expression regulates the transcriptional activation of NF-κB and AP-1. Specifically, AP-1 was primarily activated through c-JNK signaling, not p38 MAPK signaling, in LPS-stimulated pGMECs.

### Role of CD36 in regulating proinflammatory cytokine expression under LPS stimulation in pGMECs

**Figure 4:**
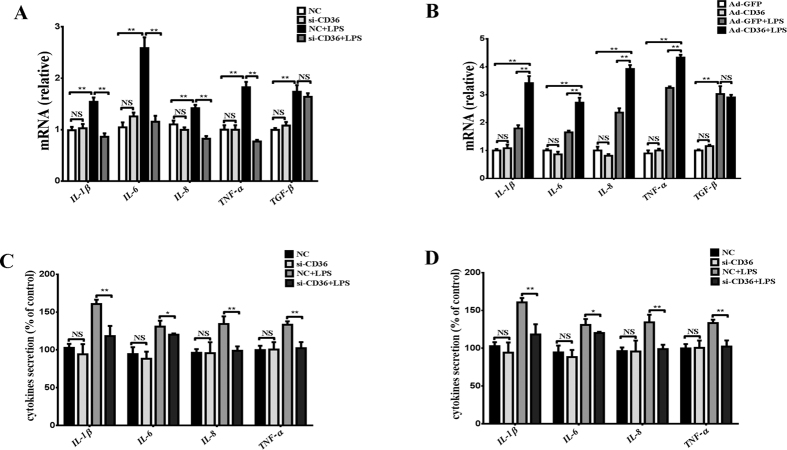
Inflammatory cytokine production is influenced by the manipulation of CD36 expression following stimulation with LPS in pGMECs. (**A**,**B**) The relative mRNA expression levels of the proinflammatory mediators were detected in CD36 knockdown pGMECs stimulated with LPS (10 μg/ml) for 12 h. Then, the cell supernatants were harvested for the analysis of TNF-α, IL-β, IL-8, and IL-6 production by ELISA. (**C**,**D**) Changes in the gene expression of the proinflammatory cytokines were evaluated in pGMECs incubated with Ad-GFP alone, Ad-GFP + LPS, or Ad-CD36 + LPS. Then, the cell supernatants were harvested for analysis of TNF-α, IL-β, IL-8, and IL-6 production by ELISA. Three replicates were evaluated in each group. The values are the mean ± SEM for three individuals. Quantitative PCR data were normalized to GAPDH, UXT, and MRPL39. The data are presented as the mean ± SEM from three experiments. *P < 0.05, **P < 0.01, and not significant (NS).

To evaluate the role of CD36 in the inflammatory response to the *E. coli*-derived ligand LPS in cells, cytokines such as IL-1β, IL-6, IL-8, TNF-α, and transforming growth factor-beta (TGF-β) were measured in CD36-depleted or CD36-overexpressed pGMECs following 12 h of stimulation with LPS. In the CD36 knockdown groups, the mRNA levels of the cytokines were significantly elevated after the cells were treated with LPS for 12 h (Fig. 4A). Compared with CD36-manipulated groups, the IL-1β, IL-6, IL-8, and TNF-α mRNA levels decreased following LPS-induced inflammation (*P* < 0.01) (Fig. 4A). However, no significant change in the mRNA levels of TGF-β (*P* > 0.05) was noted after LPS stimulation for 12 h (Fig. 4A). In the Ad-CD36 cells, the mRNA levels of IL-1β (*P* < 0.01), IL-6 (*P* < 0.01), IL-8 (*P* < 0.01), and TNF-α (*P* < 0.01) increased significantly compared with the Ad-GFP groups after treating pGMECs with LPS for 12 h (Fig. 4B). Additionally, the mRNA levels of TGF-β (*P* > 0.05) were not influenced by CD36 overexpression compared to the Ad-GFP-stimulated groups (Fig. 4B). The cytokine production levels were also similar to the mRNA levels. All cytokine levels increased after the cells were stimulated with LPS for 12 h (Fig. 4C,D). However, in the absence of CD36, IL-6, IL-8, and TNF-α, cytokine production was impaired following LPS treatment (Fig. 4C). The cytokine IL-1β (30%), IL-6 (30%), IL-8 (80%), and TNF-α (30%) levels increased notably in the Ad-CD36 groups compared to the LPS-stimulated groups (Fig. 4D). These data indicated that CD36 plays an important role in proinflammatory cytokine (IL-1, IL-6, IL-8, and TNF-α) production in pGMECs in response to the *E. coli*-derived ligand LPS.

### CD36 cooperates with TLR4 during *E. coli* internalization and phagocytosis

**Figure 5:**
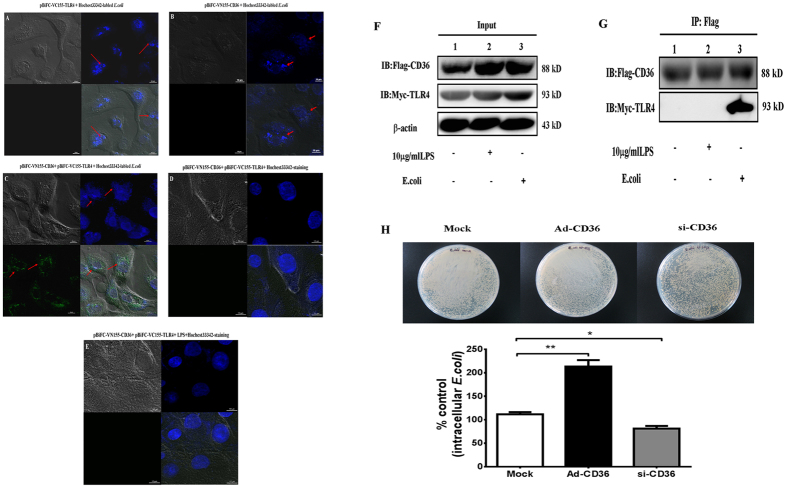
CD36 cooperates with TLR4 during *E. coli* internalization and phagocytosis. (**A**,**B**) Cells were transfected with pBiFC-VC155-TLR4 (**A**, left upper) or pBiFC-VN155-CD36 (**B**, right upper) alone, and then Hoechst 33342-labeled *E. coli* was added for 2 h at 37 °C. (**C**) Hoechst 33342-stained *E. coli* bacteria were used to stimulate cotransfection of VC-155-TLR4 and VN-155-CD36 cells for 2 h. (**D**) pGMECs were cotransfected with pBiFC-VC155-TLR4 and pBiFC-VN155-CD36 plasmids, and the cells were stained with Hoechst 33342 to visualize the nuclei. (**E**) pGMECs were cotransfected with pBiFC-VC155-TLR4 and pBiFC-VN155-CD36 plasmids, and then the cells were stimulated with LPS (10 μg/ml) and Hoechst 33342 for 2 h. The images are representative of multiple fields from three experiments. Scale bar: 10 μm. (**F**) Cells were transiently cotransfected with pef-NEO-Flag-CD36 and pef-NEO-Myc-TLR4 and then incubated with LPS for 12 h or with *E. coli* for 2 h. The cell lysates (input) were probed for Flag-CD36, Myc-TLR4, and β-actin. (**G**) Flag-CD36 was immunoprecipitated from the cell lysates using mouse anti-Flag antibody. (**H**) The cells were lysed and plated on agar after incubation with *E. coli* (10^7^ cfu/ml) in pGMEC manipulated with mock, Ad-CD36, or si-CD36 for 2 h. Equal protein amounts were immunoprecipitated (IP: Flag-CD36) using anti-Flag antibody. All data are presented as the mean ± SEM from three experiments. *P < 0.05, **P < 0.01, and not significant (NS).

The prior results suggested that CD36 cooperates with TLR4 during LPS-induced TLR4 signaling in pGMECs. Therefore, it was hypothesized that CD36 and TLR4 would also interact during bacteria recognition. To confirm that TLR4 cooperates with CD36 during *E. coli* endocytosis and phagocytosis *in vitro*, a bimolecular fluorescence complementation (BiFC) assay was used to visualize the process of pGMEC endocytosis in bacteria. pGMECs were cotransfected with pBiFC-VC155-TLR4 and pBiFC-VN155-CD36, and then Hoechst 33342-labeled *E. coli* bacteria were added for 2 h at 37 °C. No fluorescence was visualized at 488 nm after transfection with either VC-TLR4 or VN-CD36 alone or with labeled *E. coli* (Fig. 5A,B). Similarly, cotransfecting cells with VC-TLR4 and VN-CD36 and then staining them with Hoechst 33342 could not trigger green fluorescence (Fig. 5D). The only time fluorescence was visualized was when VC-TLR4, VN-CD36, and *E. coli* all existed together in the cells (Fig. 5C). The TLR4/CD36 complex at the membrane internalized the bacteria into the cytoplasm of the cell (Fig. 5C). However, TLR4 and CD36 were not combined in LPS-stimulated cells (Fig. 5E). The data showed that CD36 combined with TLR4 to form a CD36-TLR4 complex to mediate *E. coli* endocytosis in pGMECs. These data also suggested that CD36 and TLR4 might interact during the phagocytic process of cells (Fig. 5C).

From the BiFC results of this study, TLR4 is hypothesized to interact with CD36 during *E. coli* recognition and internalization in pGMECs (Fig. 5C). The vectors pef-NEO-Myc-TLR4 and pef-NEO-Flag-CD36 were cotransfected into *E. coli*-infected cells to confirm the interaction between TLR4 and CD36 in the presence of stimuli. First, cell lysates (input) were probed with Flag-tag and Myc-tag antibodies (Fig. 5F). Flag-CD36 immunoprecipitation using the Myc-TLR4 antibody showed that TLR4 interacts with CD36 in *E. coli*-infected pGMECs (Fig. 5G). Myc-TLR4 was not detected in LPS-stimulated cells (Fig. 5G). These data suggested that *E. coli*-induced infection in pGMECs might stimulate the interaction between TLR4 and CD36; however, no cooperation was shown in LPS-stimulated cells.

To understand the CD36-mediated phagocytic ability of pGMECs, the cells were treated with si-CD36 and Ad-CD36 to confirm whether manipulating CD36 expression influenced the phagocytic ability of the cells. The cells were lysed with ice-cold water and then plated on agar dishes at various dilutions for overnight incubation at 37 °C. Intracellular bacterial counts in pGMECs infected with *E. coli* in the presence of antibiotics were mainly dependent on the rate of bacteria internalization. Bacteria were incubated in Ad-CD36 cells, and the number of intracellular bacteria was greatly increased compared with the mock group (Fig. 5H). However, pGMECs deficient in CD36 had a 35% reduction in *E. coli* bacterial counts in contrast to the mock group (Fig. 5H). These data demonstrated that CD36 plays an important role in *E. coli* phagocytosis in pGMECs.

## Discussion

The *E. coli*-induced mastitis caused acinar structure disintegration and infiltration of inflammatory cells in the interstitium and acinar lumina (Fig. 1B). During this process, epithelial cells produce a variety of cytokines and initiate an inflammatory response to the noxious environmental stimuli, which links exogenous pathogen infection and endogenous immune cell activation. In *E. coli*-induced mastitis udders, the mRNA levels of TLR4 and CD36 were upregulated during the infection (Fig. 1D). Interestingly, the proteins (MyD88 and TRAF6) in the NF-κB pathway and MAPK signaling pathway were also activated in the infected tissues (Fig. 1E). In *in vivo* studies evaluating *E. coli*-induced mastitis, dairy animals showed signs of acute clinical mastitis, including somatic cell counts, decreased milk yield, udder swelling and TLR4 mRNA upregulation during the early stages of infection^31^. Mastitis studies in mouse models also found that the NF-κB and MAPK signaling pathways were activated by LPS-induced inflammation^32^,^33^. Interestingly, changes in CD36 mRNA expression were first revealed in *E. coli* mastitis.

LPS is an important virulence factor of *E. coli* that sufficiently induces an inflammatory response. In this study, LPS was used to model mastitis *in vitro*. The concentration of LPS (10 μg/ml) did not induce pGMEC apoptosis and necrosis (see Supplementary Fig. S2) but could evoke an appropriate inflammatory response (Fig. 2A). In the present study, treating pGMECs with LPS for 12 h significantly increased TLR4 mRNA levels, activated the downstream transcription factor NF-κB, and markedly elevated proinflammatory cytokines (IL-1β, IL-6, IL-8, TNF-α, and TGF-β). CD36 levels under different LPS stimulation conditions in pGMECs revealed that changes in TLR4 mRNA expression correlated with CD36 mRNA levels (Fig. 2A). Interestingly, incubation of si-CD36 pretreated pGMECs with 100 μg/ml LPS significantly increased TLR4 mRNA levels compared to the NC group possibly because a high concentration of LPS (100 μg/ml) causes TLR4 overexpression, which offsets CD36 deficiency in danger signal processing.

The TLR4-MyD88-dependent pathway was triggered by LPS and subsequently induced the production of an array of proinflammatory mediators. Based on a study by Lim *et al*., TLR4, MyD88, and CD36 participated in the oxLDL-mediated differentiation of Th17 cells in atherosclerosis^34^. Another study also demonstrated that CD36/TLR4/MyD88 mediated the production of proinflammatory cytokines in response to modified oxLDL^35^. Interestingly, impaired CD14 and CD36 expression caused by caveolin-1 deletion could attenuate TLR4 and MyD88 expression^36^. In the present study, the manipulation of CD36 expression influenced TLR4 and MyD88 expression in pGMECs. TRAF6 is an important signaling molecule that relays TLR4 signals to the NF-κB and MAPK pathways to directly modulate key cellular processes^20^. TRAF6 has been reported to be associated with Lyn kinase in LPS-stimulated mast cells^37^. However, the stimulation of endothelial cells with LPS indicated that TRAF6 interacted with Src and Fyn but not with Lyn^38^. These results demonstrated that TRAF6 interacted with Src kinases (Fyn, Lyn, and Scr) but that the interaction was dependent on cell type. Interestingly, CD36 can also mediate multiple signaling pathways primarily through the activation of Src kinase (Lyn, Fyn, Yes). Therefore, the connection between TRAF6 and CD36 could be mediated by Src kinase; however, confirmation of this hypothesis requires further testing. In this study, CD36 overexpression or deletion influenced TRAF6 expression in LPS-stimulated pGMECs.

The latest reports have demonstrated that CD36 may interact with different coreceptors and mediate multiple signaling pathways (p38-MAPK, c-JNK, and NF-κB)^12^,^13^,^39^,^40^. In this study, CD36 could influence downstream transcriptional factors (NF-κB and AP-1) in LPS-induced epithelial cells. The downstream nucleic transcription factors NF-κB and AP-1 play important roles in exogenous-induced inflammation. In a mouse model of mastitis, expression of the NF-κB p65 subunit in the mammary epithelium was confirmed in infected glands^33^. In an *in vitro* study, *E. coli* and LPS strongly activated NF-κB in bMECs^41^. In the current study, the manipulation of CD36 expression influenced MyD88, TRAF6, and NF-κB transcriptional activation in LPS-induced pGMECs, which confirmed that CD36 was involved in the MyD88-dependent signaling pathway.

The relationship between AP-1 and CD36 in *E. coli*-induced mastitis is an interesting finding. In a previous study, treatment of monocytes with oxLDL suggested that CD36 and TLR4 were involved in AP-1 activation, which could induce IL-1β production^42^. In bMECs, cells treated with heat-killed or culture supernatant of *Staphylococcus aureus* revealed that AP-1 was activated in both *S. aureus* products but not by *E. coli* or LPS-induced inflammation^24^,^43^. However, the results of this study suggested that AP-1 activation was triggered by LPS-induced inflammation. The results of these studies may differ due to the use of different types of *E. coli* in each study. AP-1 activation is regulated by MAPK (p38 and JNK) in *E. coli*-mediated inflammation^44^,^45^. This article demonstrated that c-JNK, but not p38 MAPK, was influenced by the manipulation of CD36 expression during LPS-induced activation in pGMECs. These results were similar to those obtained by Baranova *et al*., who were interested in human CD36 in LPS-induced JNK-mediated signaling^12^. LPS may trigger the activation of different MAPK kinase (MKK) pathways, such as the MKK4/7-activated c-JNK and MKK3/6-triggered p38-MAPK pathways, *via* TLR4 signaling.

When the transcriptional factors (NF-κB and AP-1) were activated, an array of proinflammatory mediators (*e.g*., IL-1β) was released to target neutrophils and mononuclear cells. In a mouse model of mastitis, IL-6 and TNF-α had high local concentrations in *E. coli* intramammary infections in wild-type mice^33^. In an *in vitro* model of mastitis, proinflammatory cytokines (*e.g*., IL-6, IL-8, and TNF-α) were highly expressed, and NF-κB was strongly activated downstream in bMECs stimulated with *E. coli* or LPS^41^,^46^. Incubating bMECs with LPS for 12 h increased the proinflammatory cytokine (IL-1β, IL-6, TNF-α, and IL-8) mRNA levels significantly compared with the control groups^47^. In *E. coli*- or LPS-induced infections, proinflammatory cytokines could affect CD36 expression^9^,^12^,^36^. According to the present study’s results, the cytokines IL-1β, IL-6, and TNF-α and chemokine IL-8, but not TGF-β, were regulated by CD36 in LPS-induced inflammation in pGMECs.

Previous research has suggested that CD36 participates in Gram-negative bacteria-induced inflammation. The scavenger B receptor CD36 is not only a transporter of fatty acids for lipid utilization but also an important component of the innate immune system, which recognizes microbial pathogens and their cell wall lipids^12^,^48^. Philips *et al*. demonstrated that the transfection of murine CD36 into human HEK293 cells greatly influenced the internalization of *E. coli* but had only a slight impact on *S. aureus* uptake^48^. Another study showed that *E. coli* and LPS were recognized by CD36, which then mediated inflammatory signaling in cells transfected with CD36^9^. Transfection of human CD36 into HeLa cells could enhance the uptake of Gram-negative bacteria, activate downstream JNK kinases and increase IL-8 production compared with the control groups^12^. The findings of this study suggested that CD36 interaction with TLR4-mediated *E. coli* phagocytosis in pGMECs, but not LPS, induced internalization possibly because *E. coli* is a complete organism that has different components, including LPS. When cells were treated with *E. coli*, a complex between CD36 and TLR4 could form possibly by variations in spatial conformation. However, stimulating cells with LPS could not induce CD36/TLR4 complex formation, which may be due to CD36 likely stimulating CD14 to present the ligand to TLR4 but not directly interact with TLR4. The results of this study were similar to those of previous studies demonstrating that CD36 served a function analogous to CD14 by cooperating with TLR4 in ligand recognition and triggering downstream cascades^49^,^50^. However, the connection between CD14 and CD36 in *E. coli*-induced inflammation is still unclear and requires further study. Additionally, whether CD36 recognizes danger signals (endogenous or exogenous) alone or in cooperation with other receptors is still controversial and must be studied further. In the current study, CD36 cooperated with TLR4 in *E. coli* phagocytosis to trigger downstream activation following LPS stimulation in pGMECs.

In conclusion, CD36 can regulate the LPS-induced inflammation process *via* the NF-κB and c-JNK signaling pathways to modulate downstream cytokine production and interplay with TLR4 in *E. coli* phagocytosis in pGMECs. This result illustrates that CD36 may serve as a new target for the treatment of *E. coli*-induced mastitis.

## Methods

### Cell culture and treatment

Mammary tissue samples were collected from healthy, lactating 2-year-old Xinong Saanen goats. The pGMECs were isolated from six healthy goats (mid-lactation) as described in a previous study^51^. Additionally, the cell culture methods were described previously^52^. Briefly, cells digested from the mammary gland of mixed samples were cultured for 2 weeks. The pGMECs were grown in DMEM/F12 medium (Invitrogen Corp., CA, USA) containing 5 mg/ml insulin, 0.25 mM hydrocortisone, 50 U/ml penicillin/ml streptomycin, 10 ng/ml epidermal growth factor-1 (EGF-1, Gibco, USA), and 10% fetal bovine serum at 37 °C in a humidified atmosphere with 5% CO_2_ 53,54. At confluence, the pGMECs were dissociated using a Trypsin-EDTA solution (0.25% Trypsin and 0.05% EDTA). At passage 1 or 2, the cells were seeded on DMEM/F12 medium in plates (Nunc, Denmark) at a density of 5 × 10^4^ cells/cm^2^ for adenovirus infection. Briefly, *E. coli* was separated and identified from four clinical *E. coli*-induced mastitis dairy goats from Shaanxi Province, China. *E. coli* separation and identification are presented in Supplementary Fig. S1 and Table S1. LPS O55:B5 was purchased from Sigma-Aldrich, USA.

### Histological analysis

Healthy and *E. coli*-infected goat mammary gland tissues were collected. The tissues were fixed in 4% paraformaldehyde at room temperature for 24 h and then embedded in paraffin. Sections (6 μm thick) were stained with hematoxylin and eosin (H&E) for histological examination. A light microscope was used to examine the sections, and the sections were imaged using a Nikon DS-U2/L2 Controller and NIS-Elements F 3.22 (Nikon Corporation, Tokyo, Japan).

### CD36 expression and manipulation and cell treatment

CD36 adenovirus was prepared for CD36 overexpression using previously published methods^55^. Goat CD36 [NM_001285578.1] was subcloned into the pAdTrack-CMV plasmid vector between the SalI and NotI (New England BioLabs, Inc., MA, USA) restriction sites to generate a pAdTrack-CMV-CD36 vector. This vector was inserted into an adenoviral vector (pAdEasy-1) to generate adenoviral plasmids in BJ5183 cells. The adenoviral plasmids linearized by PacI (New England BioLabs, Inc.) were transfected into 293A cells to generate the adenovirus pAd-CD36. The adenovirus (Ad-GFP), which was used as a positive control, was provided by Zhijie Chang (Tsinghua University, Beijing, China). Cultures of pGMECs at approximately 80% confluence were infected with Ad-GFP or Ad-CD36 at multiplicities of infection (MOIs) of 50, 100, 150, 200, or 250. The infection efficiency was determined by observing green fluorescence under inverted/phase contrast microscopy (Leica CMF-500, Germany). The infection efficiency was highest (80%) at a MOI of 100. CD36 expression was higher in the Ad-CD36-infected cells than in the Ad-GFP-infected cells at a MOI of 100 (Fig. 2C). Moreover, Ad-GFP did not affect CD36 mRNA expression compared with uninfected cells (Fig. 2C).

For CD36 knockdown, siRNA (si-CD36) was designed and synthesized by Invitrogen. si-CD36-977: sense, 5′-GUGCUAGACAUUAGCAAAUTT-3′; antisense, 5′-AUUUGCUAAUGUCUAGCACTT-3′; si-CD36-1231: sense, 5′-CCCUAUUCUUUGGCUUAAUTT-3′; antisense, 5′-AUUAAGCCAAAGAAUAGGGTT-3′. Cells treated with a nontargeting siRNA served as a NC. pGMECs were grown in plates to 80% confluence and transfected with si-CD36 and NC using Lipofectamine RNAiMAX (Invitrogen) according to the manufacturer’s instructions. After nucleofection, si-CD36-977 was found to be more efficient than si-CD36-1231 at knocking down goat CD36 (see Supplementary Fig. S3A). The time-dependent knockdown efficiencies were also detected after 24 and 48 h cell culture by real-time polymerase chain reaction (PCR) (see Supplementary Fig. S3B). pGMECs were stimulated after 24 h of infection (adenovirus) or transfection (siRNA).

### BiFC analysis

Cultured cells at 60% confluence in 6-well microscope plates were cotransfected with goat *TLR4* [JF825527.1] pBiFC-VC155 and *CD36* [NM_001285578.1] pBiFC-VN155 (I152L) together with 1.0 μg of the plasmids using Lipofectamine 2000 (Invitrogen) according to the manufacturer’s instructions^56^. After the cells were incubated for 12 h, they were washed at least three times with phosphate-buffered saline (PBS) and treated with Hoechst 33342-labeled *E. coli* in an incubator at 37 °C with 5% CO_2_ for 2 h. In the control group (not stimulated with *E. coli* and not transfected with plasmids), cell nuclei were also stained with Hoechst 33342. Then, the slides were washed with PBS to remove any unattached bacteria, fixed in 4% paraformaldehyde, and viewed immediately under a microscope. BiFC analysis was performed as described in previous studies^57^,^58^. The fluorescence complementation was observed using a Nikon confocal microscope (Nikon), and images of the middle section of the cell nucleus were taken. The Hoechst 33342 and enhanced green fluorescent protein emissions were measured at 450 and 488 nm, respectively. Image analyses for the fluorescence complementation localization were performed using the program NIS-Elements Viewer.

### Antibiotic protection assay

The endocytosis of GMECs was detected under different CD36 expression conditions (mock, Ad-CD36, and si-CD36). Before the GMECs were treated with the bacteria, they were washed with PBS. Then, the cultured cells were incubated in DMEM/F12 medium containing 10% FCS, 100 μg/ml penicillin/streptomycin, and *E. coli* (approximately, 10 bacteria/cell) in culture wells. After the cells were incubated for 2 h at 37 °C, the plates were washed with ice-cold PBS (at least three times) and lysed in ice-cold water for 40 min on ice. Lysates were plated on agar dishes at various dilutions (10^2^–10^8^ cfu/ml) and incubated overnight at 37 °C.

### Total RNA extraction and purification and quantitative real-time PCR

Total RNA from tissues and cells were separately extracted using TRIzol Reagent (Invitrogen) and an RNAprep Pure Cell Kit (Tiangen Biotech Co., Ltd., Beijing, China) according to the manufacturers’ protocols. The RNA used in the qPCR was treated with DNase (Tiangen Biotech Co., Ltd.) to remove genomic DNA contamination. cDNA was synthesized using a PrimeScript RT Kit (TaKaRa Bio, Inc., Otsu, Japan) according to the manufacturer’s instructions. Real-time quantitative RT-PCR was performed in a 96-well plate in an CFX96 sequence detector (Bio-Rad, CA, USA) using SYBR Green Reagent (SYBR Premix Ex Taq II, Perfect Real Time, TaKaRa Bio, Inc.). The primers were purchased from Sangon Biotech (Shanghai, China). The primer sequences used in this study are shown in Supplementary Table S2. The qPCR data were analyzed using the 2^−ΔΔ*Ct*^ method.

### Dual luciferase reporter gene expression assay

Cells were transiently transfected with a dual luciferase reporter gene construct of inducible firefly luciferase under the control of a NF-κB response element (pGL4.32[luc2P/NF-κB-RE/Hygro] vector, Promega, Germany) or an AP-1 response element (pGL4.44[luc2P/AP-1-RE/Hygro] vector, Promega) and a construct with constitutive *Renilla* luciferase expression. After the cells were transfected for 12 h, they were incubated with Ad-CD36 or si-CD36 for 24 h, and then LPS (10 μg/ml) was added. The cells were harvested using a commercial lysis buffer (Promega) after 48 h of transfection. The relative luciferase activity was analyzed using the Dual-Luciferase Reporter Assay (Promega) according to the manufacturer’s instructions. The relative luciferase activity of the pGL4.32[luc2P/NF-κB-RE/Hygro] and pGL4.44[luc2P/AP1 RE/Hygro] vectors was tested in GMECs before the experiment was started using Thermo Scientific Varioskan Flash (Thermo, USA) (see Supplementary Fig. S4A,B).

### Western blotting, ELISA, and immunoprecipitation analysis

After the cells were harvested, they were washed three times with cold PBS and lysed with RIPA buffer containing protease inhibitors (Solarbio, China). Protein concentrations were quantified using a BCA Protein Assay Kit (Thermo Scientific™ Pierce, USA) according to the manufacturer’s instructions. Proteins were separated on SDS-PAGE gels and transferred to nitrocellulose membranes. The blots were incubated with NF-κB p65 rabbit mAb, c-Jun rabbit mAb, p38 MAPK rabbit polyclonal antibody, TRAF-6 rabbit mAb (Cell Signaling Technology), and β-actin mouse mAb (Cwbiotech, China). The secondary antibodies used were HRP-conjugated goat anti-rabbit IgG (Cwbiotech) and HRP-conjugated goat anti-mouse IgG (Cwbiotech). Protein bands were detected using a chemiluminescent ECL Western blot detection system (Pierce) and visualized by autoradiography with a cold CCD camera (Bio-Rad). The chemiluminescent intensity was evaluated by densitometric analysis using Image-Pro Plus software 6.0.

For immunoprecipitation, pef-NEO-Myc-TLR4 and pef-NEO-Flag-CD36 were cotransfected into cells after 12 h and then incubated with *E. coli* for 2 h. The efficiencies of Myc-TLR4 and Flag-CD36 were detected after cotransfection with pGMECs for 12 h (see Supplementary Fig. S5A,B). Anti-Myc (Proteintech, China) and anti-Flag (Proteintech) antibodies were used to detect Myc-TLR4 and Flag-CD36 protein levels in the pGMECs (see Supplementary Fig. S5C). The treated cells were washed three times with ice-cold PBS and then lysed with immunoprecipitation lysis/wash buffer (Thermo). The immunoprecipitation steps were performed following the co-immunoprecipitation (Co-IP) kit (Thermo) instructions.

pGMEC secretion of cytokines (IL-1β, IL-6, TNF-α, and IL-8) was analyzed in culture supernatants after the cells were incubated with LPS (10 μg/ml) for 12 h using commercially available ELISA kits for goat cytokines (R&D Systems, USA) according to the manufacturer’s instructions. All samples and standards were measured in triplicate.

### Ethical statement

All experimental materials and protocols were approved by the Animal Care and Use Committee of Northwest A&F University. The methods were carried out in accordance with the approved guidelines.

### Statistical analysis

All of the reported results were obtained from at least three independent experiments. One-way analysis of variance (ANOVA) and *t*-test statistics were obtained using GraphPad Prism software 6.0. All of the experimental data are presented as the mean ± standard error of the mean (SEM). *P* < 0.05 was considered statistically significant.

## Additional Information

**How to cite this article**: Cao, D. *et al*. CD36 regulates lipopolysaccharide-induced signaling pathways and mediates the internalization of *Escherichia coli* in cooperation with TLR4 in goat mammary gland epithelial cells. *Sci. Rep*. **6**, 23132; doi: 10.1038/srep23132 (2016).

## Supplementary Material

Supplementary Information
