# Author Correction: CD36 regulates lipopolysaccharide-induced signaling pathways and mediates the internalization of *Escherichia coli* in cooperation with TLR4 in goat mammary gland epithelial cells

**DOI:** 10.1038/s41598-019-42156-3

**Published:** 2019-04-23

**Authors:** Duoyao Cao, Jun Luo, Dekun Chen, Huifen Xu, Huaiping Shi, Xiaoqi Jing, Wenjuan Zang

**Affiliations:** Shaanxi Key Laboratory of Molecular Biology for Agriculture, College of Animal Science and Technology, Northwest A&F University, Yangling, 712100 PR China

Correction to: *Scientific Reports* 10.1038/srep23132, published online 15 March 2016

In this Article, Figure 4D is a duplication of Figure 4C. The correct Figure 4D appears below as Figure 1.

**Figure 1:**
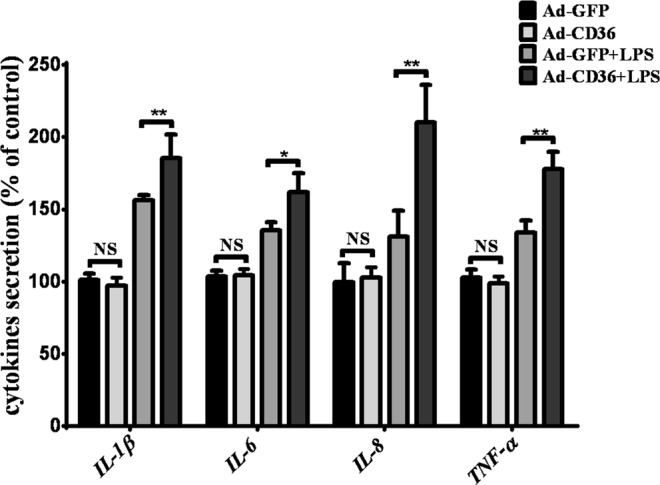
Inflammatory cytokine production is influenced by the manipulation of CD36 expression following stimulation with LPS in pGMECs. (**A**,**B**) The relative mRNA expression levels of the proinflammatory mediators were detected in CD36 knockdown pGMECs stimulated with LPS (10 μg/ml) for 12 h. Then, the cell supernatants were harvested for the analysis of TNF-α, IL-β, IL-8, and IL-6 production by ELISA. (**C**,**D**) Changes in the gene expression of the proinflammatory cytokines were evaluated in pGMECs incubated with Ad-GFP alone, Ad-GFP + LPS, or Ad-CD36 + LPS. Then, the cell supernatants were harvested for analysis of TNF-α, IL-β, IL-8, and IL-6 production by ELISA. Three replicates were evaluated in each group. The values are the mean ± SEM for three individuals. Quantitative PCR data were normalized to GAPDH, UXT, and MRPL39. The data are presented as the mean ± SEM from three experiments. *P < 0.05, **P < 0.01, and not significant (NS).

