# Supplementary material for: CD36 regulates lipopolysaccharide-induced signaling pathways and mediates the internalization of Escherichia coli in cooperation with TLR4 in goat mammary gland epithelial cells
Source: Sci Rep. 2016 Mar 15;6:23132. doi: 10.1038/srep23132 (PMC4791551; doi:10.1038/srep23132)
Supplement: Supplementary Information [file srep23132-s1.pdf]

CD36 regulates lipopolysaccharide-induced signaling pathways and mediates the internalization of *Escherichia coli* in cooperation with TLR4 in goat mammary gland epithelial cells

Authors:

Duoyao Cao , Jun Luo* , Dekun Chen , Huifen Xu , Huaiping Shi , Xiaoqi Jing, Wenjuan Zang

***Correspondence to Jun Luo [luojun1@yahoo.com]**

Tel: **+86-29-87082892**

Fax: **+86-29-87082892**

E-mail: **luojun@nwsuaf.edu.cn**

Affiliation: **Shaanxi Key Laboratory of Molecular Biology for Agriculture, College of Animal Science and Technology, Northwest A & F University, Yangling, 712100, PR China**

Supplementary Figure S1 (A) Milk samples were collected and tested for mastitis using LMT (LanZhou Mastitis Test, China). (B) Gram stain for identification of *Escherichia coli* from clinical mastitis.

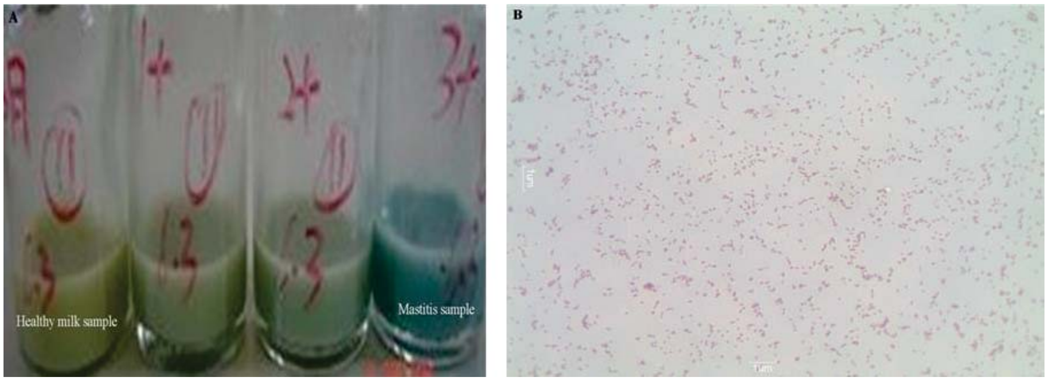

Supplementary Table S1 Biochemical identification of *Escherichia coli*

	MacConkey medium	Eosin and methylene blue medium	Triple sugar iron medium		Glucose		Lactose		Maltose		Sucrose	
			acid production	aerogenesis	acid production	aerogenesis	acid production	aerogenesis	acid production	aerogenesis	acid production	aerogenesis
1	+	+	+	+	+	+	+	+	+	+	-	-
2	+	+	+	+	+	+	+	+	+	+	-	-
3	+	+	+	+	+	+	+	+	+	+	-	-

Supplementary Figure S2 (A–D) Incubation of cells in different concentrations of LPS (1, 10, 50, and 100 μg/ml) for 12 hours. Cells were stained with Hochest33342 and PI to determine whether they induced apoptosis or necrosis. (E, F) Cells were incubated with LPS for 12

hours, and the relative mRNA levels of CD36 and TLR4 increased after stimulation. Quantitative PCR data were normalized to GAPDH, UXT, and MRPL39. Data are means ± SEM from three experiments. ***P **< 0.01, and not significant (NS).

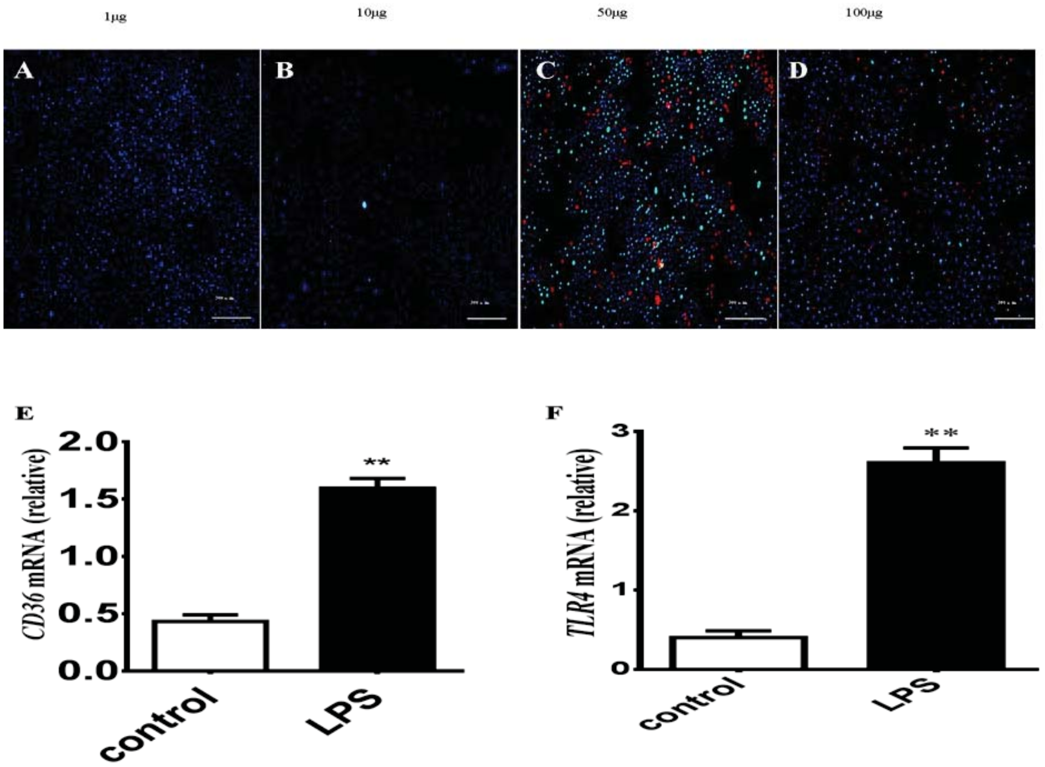

Supplementary Figure S3. Small interference RNA of CD36 in pGMECs efficiency test (A) The si-CD36-977 and si-CD36-1231 interference efficiency in pGMECs for 24 hours. (B) Changes of CD36 mRNA expression were influenced by knockdown CD36 expression in pGMECs for 24 and 48 hours. Quantitative PCR data were normalized to GAPDH, UXT, and MRPL39. Data are means ± SEM from three experiments. ***P **< 0.01, and not significant (NS).

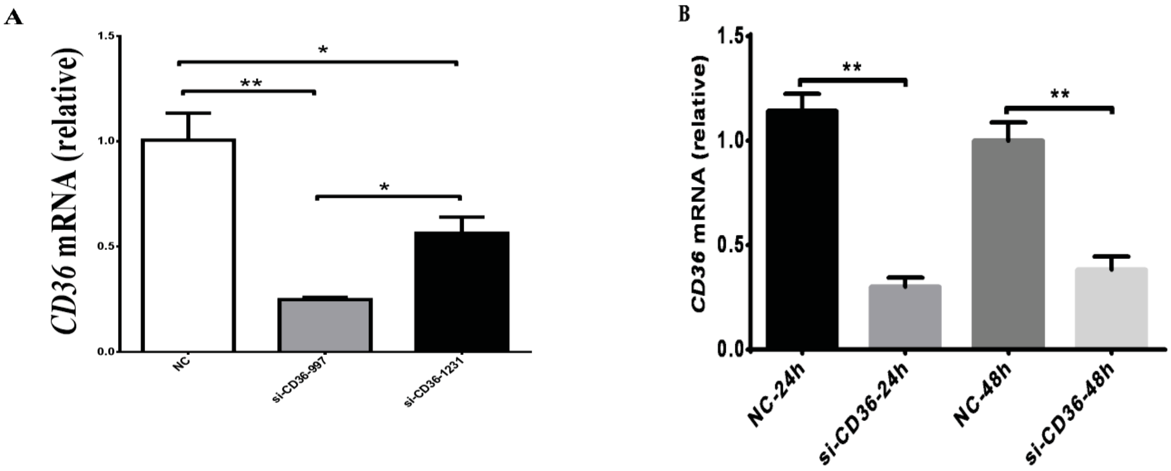

Supplementary Figure S4 Identification of the luciferase activity of pGL4-NF-κB-RE (A) and pGL4-AP-1-RE (B) vector constructs in pGMECs. Luciferase activity is compared with pGL4 luciferase vectors. Data are means ± SEM from three experiments. ******P* **< 0.001.

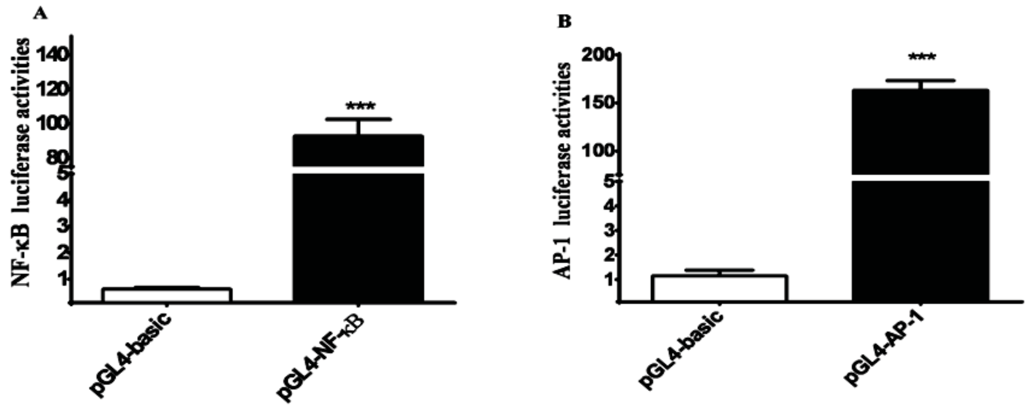

Supplementary Figure S5 The efficiency of pef-NEO-FLAG-CD36 and pef-NEO-FLAG-CD36 were cotransfected in pGMECs. CD36 (A) and TLR4 (B) mRNA levels increased significantly compared with vector groups. (C) The anti-Myc and anti-Flag were used for detection Myc-TLR4 and Flag-CD36 protein levels in pGMECs. Quantitative PCR data were normalized to GAPDH, UXT, and MRPL39. Data are means ± SEM from three experiments (***P* **< 0.01). Immunoblotting was used to test the fusion protein (Flag-CD36 and Myc-TLR4) expressions.**

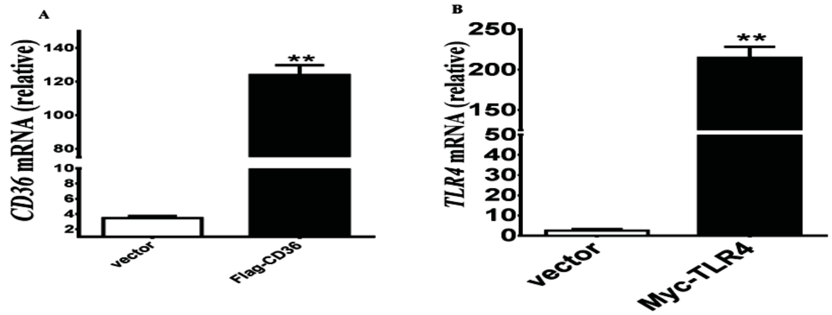

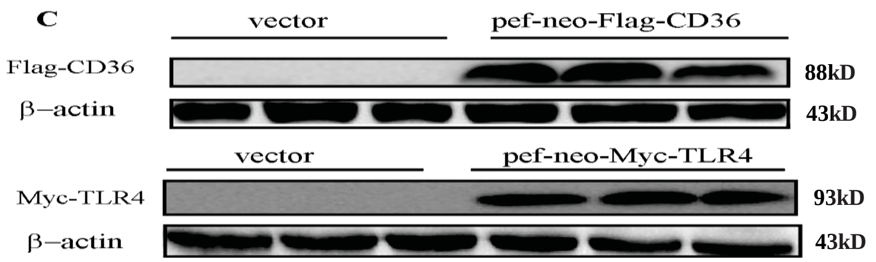

Supplement Table S2. Quantitative PCR Primers

q-PCR Primer	Sequence
GAPDH-F	GCAAGTTCCACGGCACAG
GAPDH-R	GGTTCACGCCCATCACAA
MRPL39-F	AGGTTCTCTTTTGTTGGCATCC
MRPL39-R	TTGGTCAGAGCCCCAGAAGT
UXT-F	TGTGGCCCTTGGATATGGTT
UXT-R	GGTTGTCGCTGAGCTCTGTG
IL6-F	AGATATACCTGGACTTCCT
IL6-R	TGTTCTGATACTGCTCTG
TLR4-F	AGATGGCAACACTTAGAA
TLR4-R	ATACTGAAGGCTTGGTAG
TNFa-F	TGGTTCAGACACTCAGGT
TNFa-R	CGCTGATGTTGGCTACAA
MYD88-F	CGGATGGTGGTGGTTGTCT
MYD88-R	GGAACTCTTTCTTCATTGGCTTGT
IL-1 β -F	GCGACTTCACTTTCACTTTTCACTT
IL-1 β -R	TGTGTGACCCCATAGACGGTAG
IL-8-F	AAGCTGGCTGTTGCTCTCTTG
IL-8-R	GGGTGGAAAGGTGTGGAATG
NF-Kb-F	TGGCAGCTCTTCTCAAAGCA
NF-Kb-R	GACCCCTTCATCCTCTCCATC
TGF- β -F	TTTCCGTGGGATACCGAGA
TGF- β -R	CTGTTTGCGGGGAGAGTTG
CD36-F	CGCCATAATTGACACATACA
CD36-R	CTCCGAACACAGCATAGA
